# *Myh9* Plays an Essential Role in the Survival and Maintenance of Hematopoietic Stem/Progenitor Cells

**DOI:** 10.3390/cells11121865

**Published:** 2022-06-07

**Authors:** Quanming An, Yong Dong, Yang Cao, Xu Pan, Yuan Xue, Ya Zhou, Yonggang Zhang, Feng Ma

**Affiliations:** 1 Center for Stem Cell Research and Application, Institute of Blood Transfusion, Chinese Academy of Medical Science & Peking Union Medical College (CAMS & PUMC), Chengdu 610025, China; anquanming910214@126.com (Q.A.); dong_yong@ibt.pumc.edu.cn (Y.D.); panxu@ibt.pumc.edu.cn (X.P.); battlefield2142@163.com (Y.X.); zhouya@ibt.pumc.edu.cn (Y.Z.); 2 Institute of Molecular Medicine, School of Future Technology, Beijing Key Laboratory of Cardiometabolic Molecular Medicine, Peking University, Beijing 100871, China; caoyang314@pku.edu.cn

**Keywords:** *Myh9*, HSCs, transplantation, apoptosis

## Abstract

*Myosin heavy chain 9* (*MYH9*) gene encodes a protein named non-muscle heavy chain IIA (NMHC IIA), interacting with actin and participating in various biological processes. Mutations in *MYH9* cause an array of autosomal dominant disorders, known as *MYH9*-related diseases (*MYH9*-RD). However, the role of *MYH9* in normal hematopoiesis remains largely unexplored. By using *Mx1-cre Myh9* conditional knockout mice, we established an inducible system to precisely inactivate *Myh9* function in hematopoietic cells in vivo. The results showed that deletion of *Myh9* led to severe defects in hematopoiesis, characterized by pancytopenia, drastic decreases of hematopoietic stem/progenitor cells (HSPC), and bone marrow failure, causing early lethality in mice. The defect in hematopoiesis caused by *Myh9* ablation is cell autonomous. In addition, *Myh9* deletion impairs HSPC repopulation capacity and increases apoptosis. RNA sequencing results revealed significant alterations in the expression of genes related to HSC self-renewal and maintenance, while multiple signal pathways were also involved, including genes for HSC and myeloid cell development, intrinsic apoptosis, targets of mTOR signaling, and maturity of hematopoietic cells. Our present study suggests an essential role for *Myh9* in the survival and maintenance of HSPC in normal hematopoiesis.

## 1. Introduction

Hematopoiesis is a highly orchestrated process where hematopoietic stem cells (HSC) exhibit both self-renewal and multi-lineage potentials which subsequently leads to the development of lineage-specific progenitors and their downstream mature cells [1,2]. A complex network consisting of key transcription factors regulates both self-renewal and lineage commitment of the hematopoietic cells [3,4]. Therefore, elucidating the molecular mechanisms governing the process of HSC maintenance and lineage commitment is crucial for our understanding of normal hematopoiesis and developing improved treatment of hematological diseases.

*Myosin heavy chain 9* (*MYH9*) gene, the non-muscle myosin heavy chain IIA (NMHC IIA), encodes a protein of 1960 amino acids which forms a hexameric complex including a homodimer of heavy chain (230 kDa), two regulatory light chains (20 kDa), controlling the myosin activity, and two essential light chains (17 kDa), stabilizing the heavy chain structure [5]. MYH9 interacts with actin, converting chemical energy produced by ATP hydrolysis into mechanical force [6,7,8]. It is requested in various biological processes requiring the production of force and translocation of actin cytoskeleton, such as cell migration, adhesion, division, polarization, maintenance of cell shape, and signal transduction [9,10,11,12,13]. Mutations of *MYH9* in humans lead to a syndromic, autosomal dominant disorder that is called *MYH9*-related disease (*MYH9*-RD) and characterized by congenital thrombocytopenia with giant platelets and the inclusion of NMHC IIA in the cytoplasm of neutrophils [14,15]. Ablation of *Myh9* in mice leads to embryonic death by E7.5 due to defects in cell adhesion and the visceral endoderm [16], which indicates that *Myh9* plays an essential role in early embryonic development of mice. In recent years, researchers have found that *MYH9* also regulates the maturation of erythroblasts [17] and migration of neutrophils [18], suggesting *MYH9* might exert profound effects in the hematopoietic system. These studies have provided some insights into the effect of *MYH9* in various hematopoietic lineage cells; the role of *MYH9* in regulating the function of hematopoietic stem/progenitor cells (HSPCs), however, remains largely unknown.

In the present study, we investigated the function of *Myh9* in normal hematopoiesis in vivo with a conditional *Myh9* knockout animal model. We found that the loss of *Myh9* resulted in severe defects in hematopoiesis, causing pancytopenia, BM failure, and rapid deaths in mice. The *Myh9*-deficiency mice displayed drastic reduction of HSPCs and failed to maintain normal hematopoiesis. Furthermore, loss of *Myh9* increased cell death in HSPCs and BM cells. RNA-seq analysis revealed that *Myh9* affected many hematopoietic genes and several signaling pathways involved in HSPC self-renewal and differentiation. Our findings suggest an essential role for *Myh9* in the survival and maintenance of HSPC during the process of normal hematopoiesis.

## 2. Materials and Methods

### 2.1. Mice

Floxed *Myh9 (Myh9*^fl/fl^) mice were described previously [19] and, as a generous gift from Prof. Luo jincai, *Mx1-cre* mice were purchased from Shanghai Model Organisms Center, Inc. To conditionally delete the *Myh9* floxed allele, we mated *Myh9*^fl/fl^ mice with *Mx1-cre* mice to generate *Myh9*^fl/fl^:*Mx1-cre* mice. Cre expression was induced by intraperitoneally injecting three doses of 5 mg/kg body weight polyinosinic-polycytidylic acid (poly I:C) at 5–6 weeks after birth. All mice subjected to this knockout model and other experiments were on a C57BL/6 background with littermate controls, unless stated otherwise. Genotyping primer sequences are listed in the Supplementary Table S1. All animal studies were performed in accordance with the guidelines approved by the Institutional Ethics Review Committee of Institute of Blood Transfusion (IERC-IBT).

### 2.2. Flow Cytometry

Whole BM cells were obtained by flushing femur and tibia bone with phosphate-buffered saline (PBS) containing 2% fetal bovine serum (FBS). Red blood cells were removed by lysing buffer (BD Biosciences). Cells were then washed and resuspended in PBS plus 2% FBS and stained for 30 min on ice with directly conjugated monoclonal antibodies specific for Gr-1, CD11b, CD19, CD71, Ter119. For HSC/HSPC analysis, BM cells were stained for 30min on ice with biotin conjugated lineage markers including CD3, CD4, CD8, CD11b, Gr-1, B220, Ter119. After a single wash, cells were stained for another 30 min on ice with antibodies against biotin, c-kit, sca1, CD135, CD34, CD16/32. FITC-conjugated anti-Annexin V and 7-AAD were used for apoptosis assays. All antibodies were purchased from Biolegend or eBioscience (Invitrogen). Flow cytometry was performed with Canto II (BD Biosciences) and analyzed by Flowjo software.

### 2.3. Bone Marrow Transplant Experiments

For cell autonomous BM transplantation (BMT) assay, 1 × 10^6^ BM cells from 5–6 weeks old *Myh9^fl/fl^:Mx1-cre* or littermate control mice were transplanted into lethally irradiated (2 × 550rads) C57BL/6 recipient mice by retro-orbital injection. In competitive repopulation assay, 5 × 10^5^ BM cells from uninduced *Myh9^fl/fl^:Mx1-cre* (RFP^-^) or littermate control mice (RFP^-^) were mixed with 5 × 10^5^ RFP^+^ competitor BM cells at a ratio of 1:1 and then transplanted into the irradiated RFP^-^ congenic mice. Six weeks post transplantation, recipients were administered three doses of 5mg/kg body weight poly I:C every other day to activate Cre recombinase. 

### 2.4. Complete Blood Counts and Blood Smear

Peripheral blood counts were measured by SYSMEX XT-1800i. Blood smear was stained by May-Grunwald Giemsa (MGG) as described previously [20].

### 2.5. Real-Time Quantitative PCR

Total RNA was extracted from BM cells from *Myh9^fl/fl^:Mx1-cre* and littermate control mice 10 days after poly I:C injection or FACS sorted Lin^-^Sca1^+^c-kit^+^ (LSK) cells from *Myh9^fl/fl^:Mx1-cre* and littermate control mice BM 4 days after poly I:C injection using PureLink RNA mini kit (Invitrogen). cDNA synthesis was performed using an iScript cDNA synthesis kit (Bio-rad). Real-time quantitative PCR was performed in a Bio-rad CFX96 touch machine using SYBR Green PCR master mix (Roche). The gene expression levels were normalized to beta-actin and expressed relative to the indicated reference sample. Primer sequences are listed in the Supplementary Table S1.

### 2.6. RNA-Sequencing and Data Analysis

LSK cells were harvested from *Myh9^fl/fl^:Mx1-cre* and littermate control mice 4 days after poly I:C injection. Samples were dealt with Trizol (Invitrogen). A DNA library was generated and sequenced by Novogene Co.Ltd (Beijing, China). Gene set enrichment analysis was performed as previously described [21]. Gene signatures were analyzed with Gene Set Enrichment Analysis (GSEA) software version 4.2.2.

### 2.7. Statistical Analysis

All data are shown as mean ± SD from at least three independent experiments. Statistical significance was evaluated using the Student’s *t*-test. *p*-value < 0.05 was considered significant.

## 3. Results

### 3.1. Conditional Deletion of Myh9 Leads to Pancytopenia and Fatal Bone Marrow Failure

**Figure 1:**
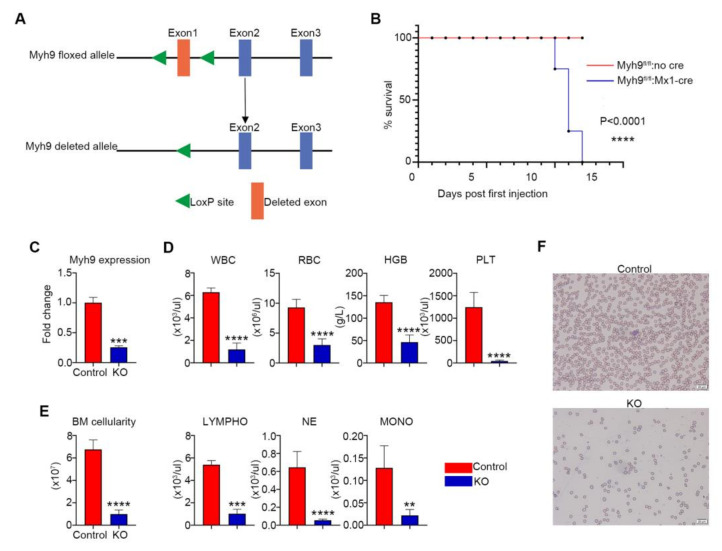
Conditional deletion of *Myh9* leads to pancytopenia and fatal bone marrow failure. (**A**) Generation of *Myh9* conditional knockout mice. The exon1 of *Myh9* is targeted by inserting two LoxP sites to create *Myh9* floxed allele. The *Myh9* floxed allele can be deleted by the expression of Cre recombinase. (**B**) Kaplan-Meier analysis showed marked decrease in survival of *Myh9^fl/fl^:Mx1-cre* mice compared with control mice after first poly I:C injection (n = 20 each group). (**C**) Real time PCR analysis for *Myh9* mRNA showed efficient deletion of *Myh9* in the BM of *Myh9^fl/fl^:Mx1-cre* after induction with poly I:C (n = 3). (**D**) Complete blood count analysis (CBC) of peripheral blood from *Myh9^fl/fl^:Mx1-cre* and control mice 10 days after injection of poly I:C, white blood cells (WBC), red blood cells (RBC), hemoglobin (HGB), platelet (PLT), lymphocyte (LYMPHO), monocyte (MONO), neutrophil (NE) (n = 5). (**E**) BM cells were significantly reduced in *Myh9^fl/fl^:Mx1-cre* mice compare to control mice 10 days after poly I:C induction (n = 5). All data are shown as mean ± SD. Student t test was used to compare two groups of mice (** *p* < 0.005, *** *p* < 0.001, **** *p* < 0.0001). (**F**) Peripheral blood smear (500×) from control and *Myh9^fl/fl^:Mx1-cre* mice at 10 days after poly I:C injection. The *Myh9^fl/fl^:Mx1-cre* blood smear showed severe anemia. Bar value *=* 20 μm.

To elucidate the physiological role of *Myh9* in normal hematopoiesis, we used the Cre-loxP system to conditionally knock out *Myh9* in hematopoietic cells in vivo (Figure 1A). *Myh9-*floxed *(Myh9^fl/fl^)* mice were crossed with *Mx1-Cre* mice to generate *Myh9^fl/fl^:Mx1-Cre* mice and the littermate mice without Cre expression were used as controls. To make *Myh9* knockout mice, 5–6 week old *Myh9^fl/fl^:Mx1-Cre* mice were injected intraperitoneally with 5 mg/kg body weight of poly I:C every other day for 3 doses, and for control mice [22]. All induced *Myh9^fl/fl^:Mx1-Cre* mice died at day10 to day12 after the first injection of poly I:C (Figure 1B). Efficiency of *Myh9* deletion was verified by real-time PCR (Figure 1C). Sequentially, we performed complete blood count (CBC) to investigate the cause of death at day10 after the first injection of poly I:C. The results showed that total white blood cell (WBC), neutrophil (NE), monocyte (MONO), lymphocyte (LYMPHO), red blood cell (RBC), hemoglobin (HGB), and platelet (PLT) counts were all significantly decreased in the peripheral blood of *Myh9^fl/fl^:Mx1-Cre* mice, as compared to control animals (Figure 1D). In addition, total BM cells were drastically decreased in *Myh9^fl/fl^:Mx1-Cre* mice (Figure 1E). As expected, knocking out of *Myh9* also caused anemia and pancytopenia in *Myh9^fl/fl^:Mx1-Cre* mice (Figure 1F), finally leading to an early lethality. These results indicated that *Myh9* was essential for hematopoiesis.

### 3.2. Myh9 Deficiency Causes Deletion of HSCs and Multiple Hematopoietic Lineage Cells 

**Figure 2:**
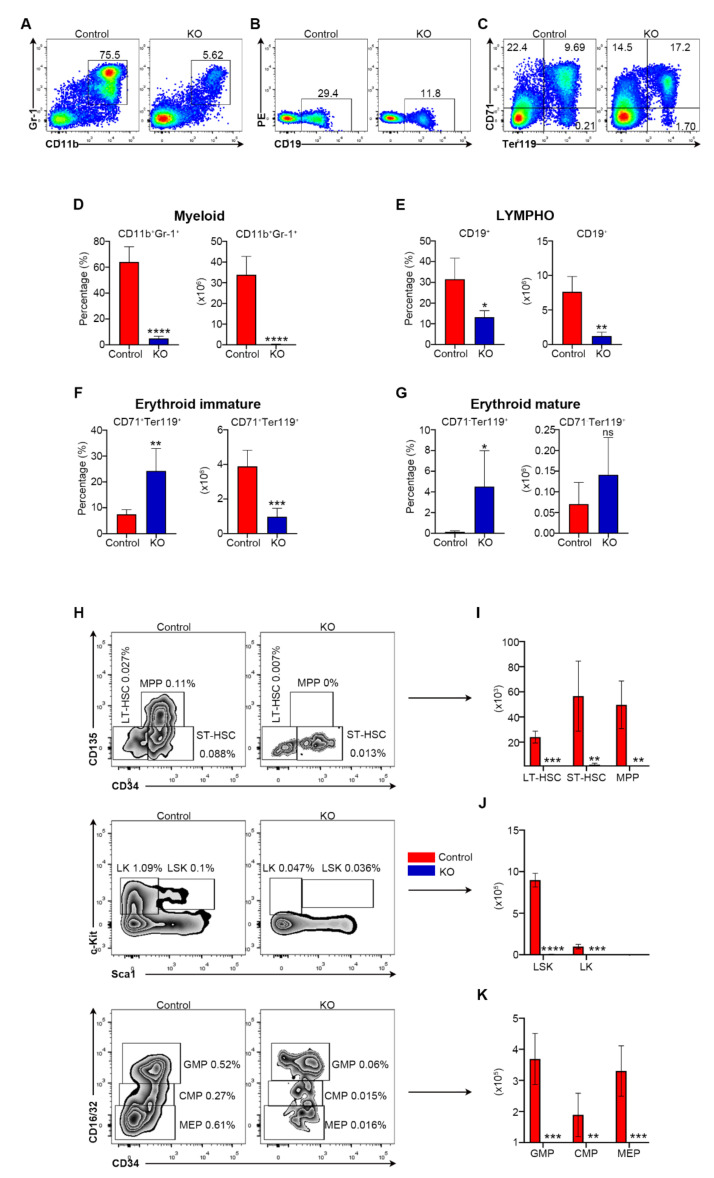
*Myh9* deficiency causes deletion of multiple hematopoietic lineages and HSC. Representative dot plots (**A**–**C**), frequencies (left), and total numbers (right) of Gr-1^+^CD11b^+^ myeloid (**D**), CD19^+^ B (**E**), CD71^+^Ter119^-^ immature erythroid cells (**F**), and CD71^-^Ter119^+^ mature erythroid cells (**G**) (n = 4–5). (**H**) Representative contour plots of flow cytometric analysis of LK(Lin^-^sca1^-^c-kit^+^), LSK (Lin^-^sca1^+^c-kit^+^), LT-HSC (Lin^-^sca1^+^c-kit^+^CD34^-^CD135^-^), ST-HSC (Lin^-^sca1^+^c-kit^+^CD34^+^CD135^-^), MPP (Lin^-^sca1^+^c-kit^+^CD34^+^CD135^+^), GMP (Lin^-^sca1^-^c-kit^+^CD34^+^CD16/32^high^), CMP (Lin^-^sca1^-^c-kit^+^CD34^+^CD16/32^low^), MEP (Lin^-^sca1^-^c-kit^+^CD34^-^CD16/32^-^). Total numbers of LT-HSC, ST-HSC and MPP (**I**); LK and LSK (**J**); GMP, CMP and MEP (**K**) in the BM of control and *Myh9^fl/fl^:Mx1-cre* mice 10 days after first poly I:C injection (n = 4). All data are shown as mean ± *SD*. Student t test was used to compare two groups of mice (* *p* < 0.05, ** *p* < 0.005, *** *p* < 0.001, **** *p* < 0.0001).

To identify the cause of the pancytopenia in *Myh9*-deficient mice, we examined hematopoiesis in their BM. Flow cytometric analysis revealed a marked decrease in the frequencies as well as numbers of mature myeloid cells (Gr-1^+^CD11b^+^), B cells (CD19^+^) in BM from *Myh9^fl/fl^:Mx1-cre* mice as compared with control mice (Figure 2A,B,D,E). Of note, *Myh9^fl/fl^:Mx1-cre* mice showed an increase in the frequencies and number of Ter119^+^ erythroid cells, particularly in mature CD71^-^Ter119^+^ erythrocytes (Figure 2C,F,G). The comparative increase in CD71^-^Ter119^+^ late-stage erythrocytes but not in CD71^+^Ter119^+^ cells might reflect a strikingly decreased total cellularity in BM and an inertness of comparatively matured erythroblasts upon induction of poly I:C (Figure 2F,G). 

The observation of multi-lineage hematopoietic abnormalities and fatal BM failure after the ablation of *Myh9* prompted us to further examine its role on HSPCs. Upon our experiments, we found the frequencies and total numbers of LSK cells, including HSCs and all multipotential stem/progenitor cell population, were drastically reduced in *Myh9^fl/fl^:Mx1-cre* BM after poly I:C injection (Figure 2H,J). Furthermore, the numbers of long-term HSCs (LT-HSC), short-term HSCs (ST-HSC), and multipotential progenitors (MPP) were markedly reduced in *Myh9^fl/fl^:Mx1-cre* mice BM when compared to control BM (Figure 2I). In the more committed LK compartments, as expected, a substantial decrease of common myeloid progenitors (CMP), granulocyte macrophage progenitors (GMP), and megakaryocyte erythroid progenitors (MEP) was detected in *Myh9^fl/fl^:Mx1-cre* mice BM as compared with controls (Figure 2K). These findings suggest that deficiency of *Myh9* leading to a whole decrease of hematopoietic stem/progenitor and committed lineage cells, thus leading to the BM failure and pancytopenia in peripheral blood. Namely, loss of *Myh9* caused severe defects in normal hematopoietic development.

### 3.3. Myh9 Function in Hematopoiesis Is Hsc Intrinsic

**Figure 3:**
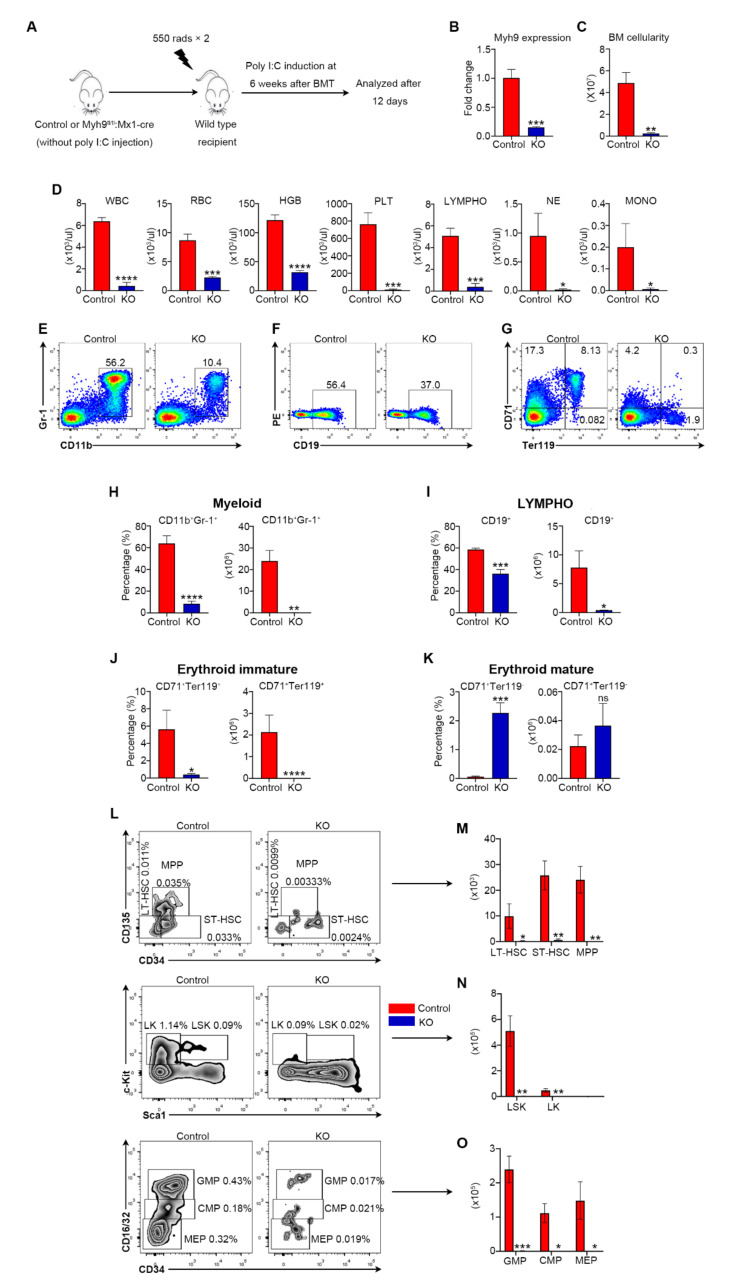
*Myh9* function in hematopoiesis is cell autonomous. (**A**) Schematic diagram of bone marrow transplantation strategy. 1 *×* 10^6^ BM cells were harvested from uninduced *Myh9^fl/fl^:Mx1-cre* and control mice and transplanted into lethally irradiated wild type C57BL/6 recipient mice. Six weeks after bone marrow transplantation, three doses of poly I:C were injected to induce the deletion of *Myh9* in donor-derived hematopoietic cells. Mice were analyzed 12 days after first poly I:C injection. (**B**) Real time PCR analysis showed deletion of *Myh9* in the BM of *Myh9^fl/fl^:Mx1-cre* recipient mice after induction of poly I:C (n = 3). (**C**) BM cellularity was significantly reduced in *Myh9^fl/fl^:Mx1-cre* recipient mice compared with control mice 12 days after poly I:C injection (n = 4). (**D**) Peripheral blood counts were assessed at 12 days after poly I:C induction in control and *Myh9^fl/fl^:Mx1-cre* BMT mice (n = 4). Representative dot plots (**E**,**F**,**G**), frequencies, and total numbers of Gr-1^+^CD11b^+^ myeloid (**H**), CD19^+^ B cells (**I**), CD71^+^Ter119^-^ immature erythroid cells (**J**), and CD71^-^Ter119^+^ mature erythroid cells (**K**) (n = 4–5). (**L**) Representative contour plots of flow cytometric analysis of LK, LSK, LT-HSC, ST-HSC, MPP, GMP, CMP, MEP. Total numbers of LK and LSK (**M**); LT-HSC, ST-HSC and MPP (**N**); GMP, CMP, and MEP (**O**) in the BM of control and *Myh9^fl/fl^:Mx1-cre* BMT mice 12 days after poly I:C injection (n = 4). All data are shown in bar graphs as mean ± SD. Student t test was used to compare two groups of mice (* *p* < 0.05,** *p* < 0.005,*** *p* < 0.001,**** *p* < 0.0001).

Since *Mx1-Cre* expression is not limited in hematopoietic cells, the observed HSC phenotypes in *Myh9^fl/fl^:Mx1-cre* mice may be caused by the dysfunction of multiple tissues resulting from the loss of *Myh9*. To verify that the loss of HSC/progenitors in *Myh9* KO animals is HSC intrinsic, we transplanted uninduced control (*Myh9^fl/fl^:no cre*) and *Myh9^fl/fl^:Mx1-cre* mice into lethally irradiated WT C57BL/6 mice. Six weeks after transplantation, recipients were injected with poly I:C to induce the deletion of *Myh9* (Figure 3A). As a result, all the recipients reconstituted with *Myh9^fl/fl^:Mx1-cre* BM cells became moribund and died within 12–14 days after first injection of poly I:C. Analysis by real time PCR showed a significant decrease in *Myh9* mRNA levels in BM cells upon poly I:C induction in the recipient animals (Figure 3B). Deletion of *Myh9* in the recipient animals resulted in marked decrease in BM cellularity (Figure 3C) and PB counts (pancytopenia) (Figure 3D). Furthermore, *Myh9* deletion also resulted in a drastic decrease in myeloid, erythroid, and B cell precursor (Figure 3E–K) as well as HSCs and hematopoietic progenitor cells (Figure 3L–O) in the BM of recipient animals. These results demonstrated that the hematopoietic defect was caused by the loss of *Myh9* on HSC intrinsically.

### 3.4. Loss of Myh9 Results in Impaired Repopulation of HSCs

**Figure 4:**
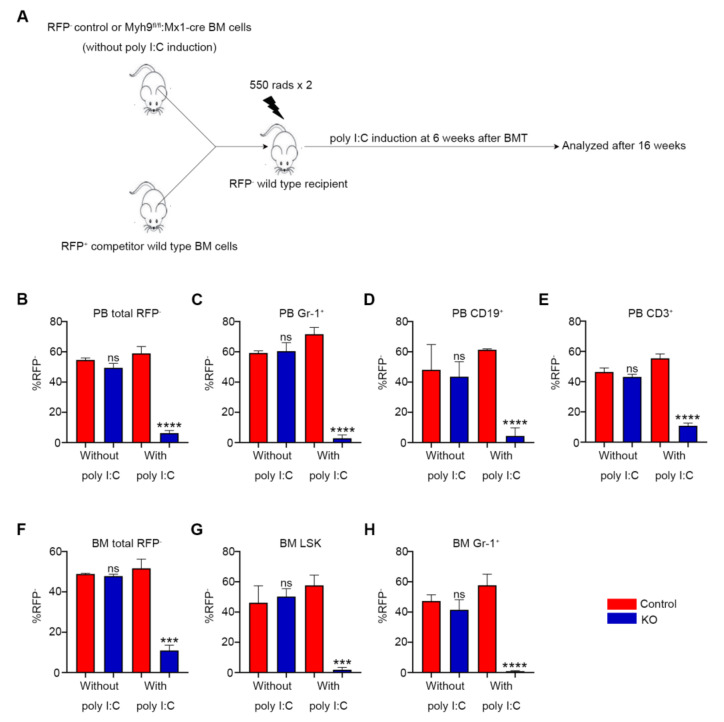
*Myh9* deficiency impairs repopulation capacity of HSC. (**A**) Schematic diagram of bone marrow competitive reconstitution assay. BM cells (5 *×* 10^5^) from uninduced RFP^-^ *Myh9^fl/fl^:Mx1-cre* or littermate control mice were mixed with RFP^+^ wild type mice BM cells (5 *×* 10^5^) at a 1:1 ratio and transplanted into lethally irradiated RFP^-^ recipient mice. Six weeks after BMT, recipient mice were treated with three doses of poly I:C to delete *Myh9* after hematopoietic reconstitution. The recipient mice were analyzed at 6 weeks after BMT and 16 weeks after poly I:C injection. Bar graphs show the percentage of donor derived RFP^-^ cells (**B**), percentage of RFP^-^Gr-1^+^ myeloid cells (**C**), percentage of RFP^-^CD19^+^ B cells (**D**), percentage of RFP^-^CD3^+^ T cells (**E**) in the peripheral blood of recipient mice at 6 weeks after BMT (without poly I:C) and 16 weeks after poly I:C injection (with poly I:C). The percentage of donor derived RFP^-^ cells (**F**), RFP^-^LSK cells (**G**), RFP^-^Gr-1^+^ myeloid cells (**H**) in the BM of recipient mice at 16 weeks after poly I:C injection (n = 3–4). All data are shown as mean ± SD. Student t test was used to compare two groups of mice (*** *p* < 0.001, **** *p* < 0.0001).

To further examine the ability of *Myh9*-deficient HSCs in hematopoietic reconstitution, competitive repopulation assays were performed. We transplanted RFP^-^ BM cells from uninduced control (*Myh9^fl/fl^:no cre*) and *Myh9^fl/fl^:Mx1-cre* mice together with RFP^+^ competitor BM cells at a ratio of 1:1 into lethally irradiated RFP^-^ recipient animals (Figure 4A). Six weeks after the transplantation, recipients were sacrificed to harvest BM and PB cells and the percentages of RFP^-^ and RFP^+^ were analyzed. The recipients reconstituted with *Myh9^fl/fl^:Mx1-cre* BM cells have a similar RFP^-^ ratio when compared with the controls, indicating the success of transplantation (Figure 4B-H, without poly I:C). Subsequently, three doses of poly I:C were injected. As expected, RFP^-^ *Myh9^fl/fl^:Mx1-cre* mice derived BM cells were completely unable to compete with co-transplanted RFP^+^ wild type cells after poly I:C injection. At 16 weeks after poly I:C induction, *Myh9^fl/fl^:Mx1-cre* mice derived RFP^-^ myeloid, B and T cells were almost abolished in the PB (Figure 4B-E, with poly I:C) of the recipient animals. Meanwhile, RFP^-^Gr-1^+^ cells, LSK cells from *Myh9^fl/fl^:Mx1-cre* mice were almost undetectable in the BM of recipients, whereas approximately 60% LSK cells in recipients that had received control BM cells were RFP^-^ (Figure 4F–H, with poly I:C). These data proved that the deletion of *Myh9* led to severe functional impairment in HSCs. Because of the severe BM failure observed in primary and BMT mice, we were not able to further explore the self-renewal capacity of *Myh9*-deficent HSCs by secondary transplantation.

### 3.5. Loss of Myh9 Leads to Apoptosis of Hematopoietic Stem/Progenitor Cells 

**Figure 5:**
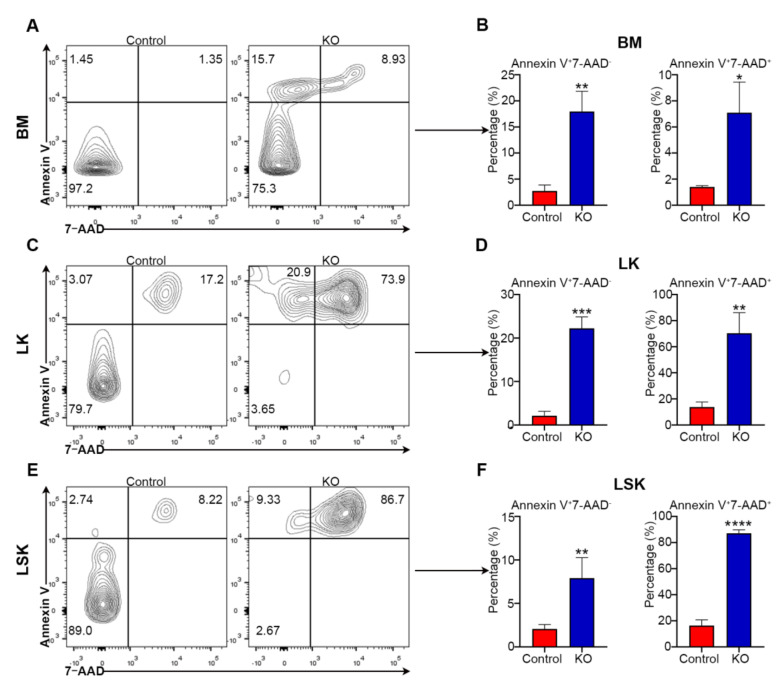
Loss of *Myh9* leads to apoptosis of hematopoietic stem/progenitor cells. Representative dot plots and bar graphs of apoptotic cells from BM (**A**,**B**), LK (**C**,**D**), and LSK (**E**,**F**) cells of control and *Myh9^fl/fl^:Mx1-cre* mice 8 days after first poly I:C injection (n = 3). All data are shown as mean ± SD. Student t test was used to compare two groups of mice (* *p* < 0.05,** *p* < 0.005,*** *p* < 0.001,**** *p* < 0.0001).

Since *Myh9* deletion caused rapid decrease in hematopoietic cells of multiple lineages in the BM, we wondered whether the deletion of *Myh9* induced apoptosis in hematopoietic cells. We performed Annexin V and 7-AAD staining to identify apoptotic cells in BM. The frequency of total BM cells undergoing apoptosis was significantly higher in *Myh9^fl/fl^:Mx1-cre* mice 8 days after poly I:C administration as compared to control mice (Figure 5A,B). Consistently, *Myh9* deletion also resulted in markedly increased apoptotic frequency of LK cells (Figure 5C,D) and more immature LSK cells (Figure 5E,F). Overall, these data suggest that loss of *Myh9* led to apoptosis in HSPCs.

### 3.6. Myh9 Deletion Alters Expression of Genes Responsible for HSC Maintenance

**Figure 6:**
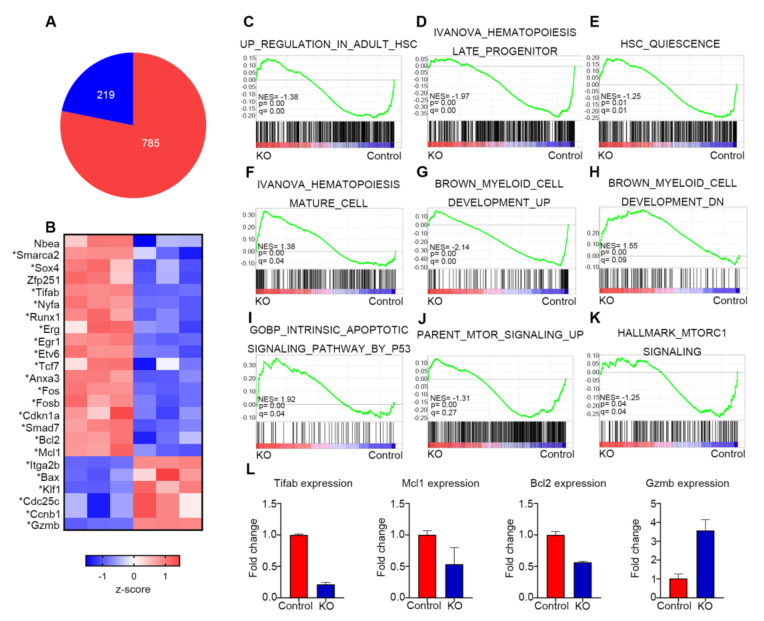
*Myh9* deletion alters expression of genes responsible for HSC maintenance. (**A**) Pie chart showing the numbers of differentially expressed genes in *Myh9*-deficient LSK cells 4 days after first poly I:C injection. (**B**) Heat map showing interested genes involved in regulation of HSC self-renewal, survival, and maintenance. Genes with * are significantly up- or down- regulated. (**C**–**K**) Gene set enrichment analysis showed significant alterations of genes related to hematopoietic stem cells, myeloid cells, survival, and mTOR signal pathways in *Myh9^fl/fl^:Mx1-cre* LSK cells compared with control LSK cells 4 days after first poly I:C injection. (**L**) Expression level of genes from selected gene sets were confirmed by real time PCR (n = 3). Data are shown as mean ± SD.

To gain insight into molecular mechanisms of the severe hematopoietic defects observed in the *Myh9*-deficient mice, transcriptome profiling was conducted by RNA-seq using sorted LSK cells from the BM of control and *Myh9^fl/fl^:Mx1-cre* mice with three independent LSK cell sets at d4 post the first injection of poly I:C. The RNA-seq data revealed a significantly altered expression (>or <2-fold, *p* < 0.05, respectively) of 785 (566 upregulated and 219 downregulated) genes in *Myh9*-deficienct LSKs when compared with control LSKs (Figure 6A). Furthermore, *Myh9* deletion resulted in downregulation of many important transcription factors required for maintenance of HSCs (*Fos, Fosb, Egr1, Erg, Runx1*, *Etv6*, and *Tcf7*) and HSPC self-renewal (*Nbea, Smarca2, Sox4, Zfp251, Tifab, Smad7*). Knockdown of these genes has been shown to impair HSPC maintenance, self-renewal, and BM reconstitution ability [23,24,25]. We also found anti-apoptotic genes (*Bcl1, Mcl1*) were downregulated and pro-apoptotic gene (*Bax*) was upregulated after *Myh9* deletion. Our results show that regulators of the cell cycle (*cdc25c* and *ccnb1*) and megakaryocyte-erythroid progenitor lineage (*Itga2b* and *Klf1*) were among those upregulated (Figure 6B).

To identify specific gene sets that were altered in *Myh9*-deficienct LSKs, gene set enrichment analysis (GSEA) was performed for the gene expression profiles of *Myh9* deficient LSKs with previously reported hematopoiesis-related gene sets. First, we compared our data with genes enriched in adult HSC and hematopoiesis progenitors [26], and these signatures were significantly downregulated in *Myh9* deficient LSKs but were enriched in control LSKs (Figure 6C,D). Second, we compared our data with the genes that were commonly upregulated in adult quiescent HSCs [27]. Again, this quiescence signature was downregulated in *Myh9* deficient LSKs (Figure 6E). Third, we used hematopoiesis mature related gene set [28] (Figure 6F); this mature signature was highly enriched in *Myh9* deficient LSKs but downregulated in control LSKs. Altogether, these results indicated that loss of *Myh9* perturb the homeostasis of HSC resulted in impairing HSC maintenance. Besides, we found that myeloid cell development gene set [29] was downregulated and intrinsic apoptotic signaling pathway was upregulated in *Myh9* deficient LSKs (Figure 6G–I), consistent with markedly increased apoptosis and decreased neutrophils as observed in *Myh9* deficient mice.

Interestingly, result of GESA showed that mammalian target of rapamycin (mTOR) related pathway gene sets were downregulated in *Myh9* deficient LSKs (Figure 6J,K). Previous studies demonstrated that the mTOR signal pathway was indispensable for HSC self-renewal and loss of mTOR result in pancytopenia in mice [30]. These results suggested that the pancytopenia caused by *Myh9* deletion in mice might be partly due to the impaired mTOR signal pathway. 

To confirm RNA sequencing results, four genes (*Gzmb*, *Mcl1*, *Bcl2*, *Tifab*), which had well-established functions related to HSCs, were selected. As expected, *Mcl1, Bcl2, Tifab* were all downregulated and *Gzmb* was upregulated in RT-PCR analysis (Figure 6L).

## 4. Discussion

*MYH9* has been reported to play critical roles in processes that are fundamental to sustaining development of the organism. *Myh9* knockouts showed an embryonic death around E7.5, a stage that various organs were being developed, including the initiation of the first hematopoietic wave [31]. In murine blood cells, mutation of *MYH9* could conduct severe megathrombocytopenia and functional damage for cell adhesion in mature neutrophils and affected morphology of RBC through association with F-actin [14,15,17,18]. In humans, mutation in *MYH9* results in hematopoietic defects such as macrothrombocytopenia, NMHC ⅡA aggregation in neutrophil granulocytes, which makes it important to study the role of *MYH9* in hematopoiesis. However, an understanding of the function of *Myh9* is still poor, partially because of the embryonic lethality and wide-range damage for *Myh9* knockout model mouse. Establishing a precise and inducible deficiency of *Myh9*, especially in vivo, should uncover its unknown function on hematopoiesis and provide new insight for developing treatment of *MYH9*-RD. Although the establishment of a mouse model for 3 types of *Myh9* mutation (R702C, D1424N, and E1841K) that mimic counterparts in human diseases was reported, it was designed to aim at the effect of *Myh9* mutations on granulocytes or thrombocytes by using knock-in heterozygous mice [32,33]. The effects raised by dysfunction of whole *Myh9* gene on hematopoiesis were not addressed, particularly on HSCs. 

In this study, we used a conditional *Myh9* knockout mouse model to elucidate the role of *Myh9* in normal adult hematopoiesis in vivo. Our data demonstrated that deletion of *Myh9* resulted in a comprehensive damage of hematopoiesis, including pancytopenia in peripheral blood, loss of HSPCs with a marked decrease in BM cellularity, and finally caused BM failure and early lethality in *Myh9* deficient mice. By using a Cre-LoxP system to inactivate *Myh9* and an in vivo inducible system, our method could ensure that we fulfil the aim to precisely analyze the loss of function of *Myh9* on hematopoietic cells. Collectively, our results suggest an essential role for *Myh9* in normal hematopoiesis.

After initial poly I:C injection for 12 days, the recipient mice reconstituted with *Myh9^fl/fl^:Mx1-cre* BM cells showed a drastic reduction of HSCs, HPCs, and other hematopoietic precursor cells in BM, along with a typical pancytopenia in peripheral blood and, finally led to death. Such a fast lethality in both primary and BM tranplantation recipient mice might be due to the effect of *Myh9* deletion on more differentiated cells rather than the consequential damage on HSC/HPCs. Although the effect of *Myh9* on mature cells might be the main cause of the rapid death of mice, the loss of *Myh9* on HSC/HSPCs also made it impossible to replenish the gross loss of differentiated cells, which further exacerbates the phenotype. Upon our observation, *Myh9* deficient HSCs were functionally hampered to develop into mature hematopoietic precursors and severely defective in repopulation capacity. Particularly, *Myh9* deficient HSCs were completely unable to compete with co-transplanted RFP^+^ wild type HSCs. The failure of *Myh9* deficient HSCs to reconstitute the hematopoietic system in non-competitive and competitive BMT assays also revealed that loss of *Myh9* intrinsically impaired the self-renewal capacity of HSCs, making them unable to maintain long-term hematopoiesis. In line with this rapid reduction of HSCs, Annexin V and 7-AAD staining showed that deletion of *Myh9* significantly increased the frequency of apoptosis in HSCs and HPCs, which might contribute to the loss of hematopoietic homeostasis. Our results strongly suggest that *Myh9* was indispensable in maintaining normal function of HSCs, while deficiency of *Myh9* would greatly damage the development and differentiation of hematopoietic cells. 

Recently, Mx1-Cre transgenic mice have been widely used to knockout target genes in the hematopoietic system. Of note, as an inducer of type I IFN signaling, poly I:C used in this study might push the stem cells into cycling and back, resulting in a proliferation of quiescent HSC pool as compared with uninduced wild type controls [34]. Thus, the system we used by treating flox mice with poly I:C in either *Myh9*-deletion or control mice could not rule out this possibility. In addition, the combination of the pro-proliferative and inflammatory stimulus with a certain targeted deletion could cause different effects than the targeted deletion in an unstimulated hematopoietic system. Therefore, the inflammatory stimulus of poly I:C in our model might also play a role in rapidly deriving lethal hematopoietic phenotype of *Myh9* ablation, as was previously observed with other molecules [35]. Use of alternative inducible promoters was, however, beyond the scope of this work.

RNA-seq analysis *Myh9*-deleted LSK cells revealed that many genes responsible for HSC regulation were altered, impairing HSC self-renewal, maintenance, and survival, including *Nbea, Smarca2, Sox4, Zfp251, Fos, Fosb, Egr1, Erg, Runx1, Etv6*, and *Tcf7* (Figure 6). Moreover, we observed changes in the expression of *Tifab* and *Gzmb* in *Myh9*-deleted LSK cells. *Tifab* (TRAF-interacting protein with forkhead-associated domain B) has been manifested to regulate hematopoiesis through Toll-like receptor (TLR)-TRAF6 complex and loss of *Tifab* induces BM failure and myelodysplastic syndrome (MDS) [36]. In addition, the increased expression of *Gzmb* (Figure 6B) might also contribute to HSPC damage, as a blockade of *GzmB* can improve HSC reconstitution, whereas enhancement of *GzmB* results in an increased apoptosis in HSPC [37]. Interestingly, upon sequencing analysis of *Myh9*-deficient LSK cells, we found that one prosurvival signaling gene set critical for mTOR function was markedly downregulated, while the other one that is important for intrinsic apoptotic signaling pathway by *p53* was markedly upregulated (Figure 6). Since the loss of mTOR in hematopoiesis was sufficient to cause pancytopenia and impairment of HSC homeostasis [38] and *P53* pathway was activated by DNA damage and played an important role in regulating HSC quiescence, self-renewal, and apoptosis, our results indicated that defects observed in *Myh9*-deficient mice might cause a perturbation of multiple genes and signaling pathways related to HSC maintenance. Recent reports have shown that *Myh9* is crucial for maintaining stemness of lung cancer cells and promoting tumorigenesis via activating mTOR signals [39,40], suggesting *Myh9* may exert a profound function on stem cells.

Although our study elucidated the role of *Myh9* in normal hematopoiesis in mice, it did not simply represent the role of *MYH9* in human *MYH9*-RD. In humans, most *MYH9*-RD was caused by point mutations and mouse models mimicking *MYH9*-RD exhibited similar results to humans [32,33]. However, in our present study, *Myh9* was knocked out in the hematopoietic system by Cre-Loxp, resulting in a complete loss of NMII A molecular rather than by making point mutation. As the result, this induced defect of *Myh9* in the hematopoietic system might reveal more comprehensive damage in hematopoiesis.

## 5. Conclusions

By using a *Myh9^flox/flox^:Mx1-Cre* model we measured the effect of *Myh9* deletion in vivo and clarified its unreported function on hematopoiesis. Our findings suggest that *Myh9* played an important role in the maintenance of adult HSCs. The inducible *Myh9* knockout mouse model offered a useful tool to explore the role of *Myh9* on different hematopoietic lineages and tissue development in vivo. The in vivo transplantation results strongly suggested that *Myh9* was indispensable in maintaining normal function of HSCs and HPCs, while deficiency of *Myh9* would greatly damage the processes of development and differentiation of hematopoietic cells. Nevertheless, the data we accumulated in the present study could only speculate a possible mechanism for how *Myh9* impaired hematopoiesis in mice as we summarized in Supplementary Figure S1. Since other reports and ours showed a comprehensive effect of *Myh9* on hematopoiesis, further research should utilize more subtle methods, such as single cell sequencing, to focus on understanding the molecular mechanisms that control *Myh9* in the regulation of normal and diseased hematopoiesis.

## Data Availability

The RNA-sequencing datasets reported in this paper can be found at GEO: GSE198134.

## Supplementary Materials

The following supporting information can be downloaded at: https://www.mdpi.com/article/10.3390/cells11121865/s1, Figure S1: A possible mechanism of the deletion of *Myh9* in the murine hematopoietic system; Table S1: Primers used for genotyping and qRT-PCR.
