# Supplementary material for: Myh9 Plays an Essential Role in the Survival and Maintenance of Hematopoietic Stem/Progenitor Cells
Source: Cells. 2022 Jun 7;11(12):1865. doi: 10.3390/cells11121865 (PMC9221478; doi:10.3390/cells11121865)
Supplement: Supplementary file 1 [file cells-11-01865-s001.zip › cells-1645079-supplementary/Supplementary material/Supplementary Materials Figure S1.pdf]

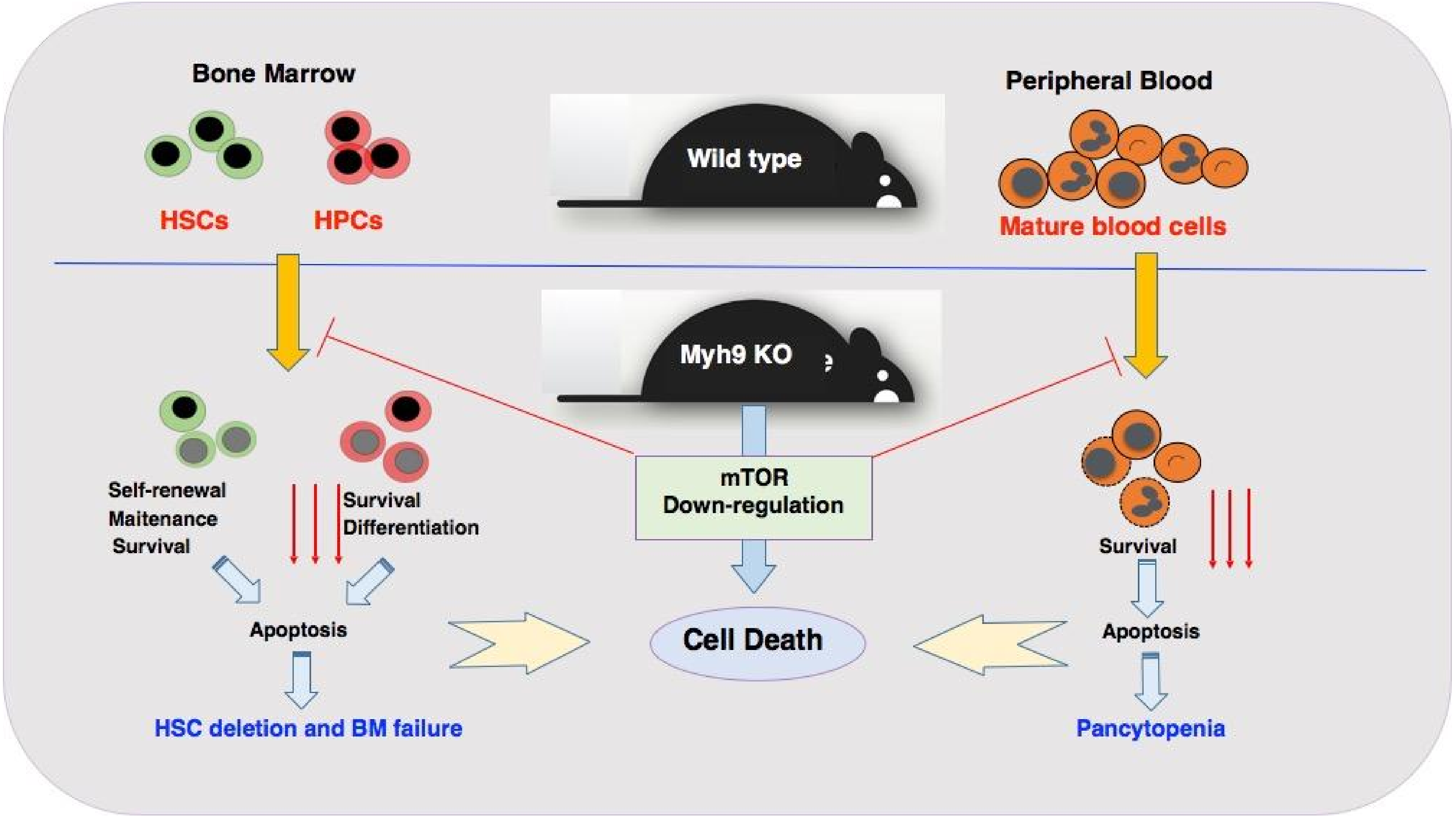

Supplementary Figure 1. A possible mechanism of the deletion of Myh9 in the murine hematopoietic system. Loss of Myh9 in mature blood cell leads to increased apoptosis and pancytopenia in peripheral blood through mTOR signaling pathway, which might be the main cause of rapid death of mice. This phenotype is further exacerbated by HSC deletion and BM failure caused by dysfunction of HSPC self-renewal, maintenance and survival via impaired mTOR signaling pathway in Myh9 deficient mice.
