# Supplementary material for: Myh9 Plays an Essential Role in the Survival and Maintenance of Hematopoietic Stem/Progenitor Cells
Source: Cells. 2022 Jun 7;11(12):1865. doi: 10.3390/cells11121865 (PMC9221478; doi:10.3390/cells11121865)
Supplement: Supplementary file 1 [file cells-11-01865-s001.zip › cells-1645079-supplementary/Supplementary material/Supplementary Materials Table S1.pdf]

Supplementary Materials:

Supplementary Table S1：Primers used for genotyping and qRT-PCR.

Mouse Myh9 genotyping Forward	TGTCATTAGTGTCCTGAGAGCAG
Mouse Myh9 genotyping Reverse	CAGGCATGGAGTTTGTGATG
Mouse Mx1-cre genotyping Forward	GTGAGTTTCGTTTCTGAGCTCC
Mouse Mx1-cre genotyping Reverse	CGGTTATTCAACTTGCACCA
Mouse Tifab RT-PCR Forward	GTTGGGCGAGGACAGAACAC
Mouse Tifab RT-PCR Reverse	GTCAGCACCTTGAGGCAGA
Mouse Mcl1 RT-PCR Forward	AAAGGCGGCTGCATAAGTC
Mouse Mcl1 RT-PCR Reverse	TGGCGGTATAGGTCGTCCTC
Mouse Bcl1 RT-PCR Forward	GCGTACCCTGACACCAATCTC
Mouse Bcl1 RT-PCR Reverse	CTCCTCTTCGCACTTCTGCTC
Mouse Gzmb RT-PCR Forward	CCACTCTCGACCCTACATGG
Mouse Gzmb RT-PCR Reverse	GGCCCCCAAAGTGACATTTATT
